# CD44: A Multifunctional Mediator of Cancer Progression

**DOI:** 10.3390/biom11121850

**Published:** 2021-12-09

**Authors:** Malak Hassn Mesrati, Saiful Effendi Syafruddin, M. Aiman Mohtar, Amir Syahir

**Affiliations:** 1 Nanobiotechnology Research Group, Department of Biochemistry, Faculty of Biotechnology and Biomolecular Sciences, Universiti Putra Malaysia, Serdang 43400 UPM, Selangor, Malaysia; malak.hassan.rh@gmail.com; 2 UKM Medical Molecular Biology Institute (UMBI), Universiti Kebangsaan Malaysia, Cheras, Kuala Lumpur 56000, Malaysia; effendisy@ppukm.ukm.edu.my (S.E.S.); m.aimanmohtar@ppukm.ukm.edu.my (M.A.M.); 3 UPM-MAKNA Cancer Research Laboratory, Institute of Bioscience, Universiti Putra Malaysia, Serdang 43400 UPM, Selangor, Malaysia

**Keywords:** CD44, regulation, tumourigenesis, signalling pathways, prognosis, therapeutic targeting

## Abstract

CD44, a non-kinase cell surface transmembrane glycoprotein, has been widely implicated as a cancer stem cell (CSC) marker in several cancers. Cells overexpressing CD44 possess several CSC traits, such as self-renewal and epithelial-mesenchymal transition (EMT) capability, as well as a resistance to chemo- and radiotherapy. The CD44 gene regularly undergoes alternative splicing, resulting in the standard (CD44s) and variant (CD44v) isoforms. The interaction of such isoforms with ligands, particularly hyaluronic acid (HA), osteopontin (OPN) and matrix metalloproteinases (MMPs), drive numerous cancer-associated signalling. However, there are contradictory results regarding whether high or low CD44 expression is associated with worsening clinicopathological features, such as a higher tumour histological grade, advanced tumour stage and poorer survival rates. Nonetheless, high CD44 expression significantly contributes to enhanced tumourigenic mechanisms, such as cell proliferation, metastasis, invasion, migration and stemness; hence, CD44 is an important clinical target. This review summarises current research regarding the different CD44 isoform structures and their roles and functions in supporting tumourigenesis and discusses CD44 expression regulation, CD44-signalling pathways and interactions involved in cancer development. The clinical significance and prognostic value of CD44 and the potential of CD44 as a therapeutic target in cancer are also addressed.

## 1. Introduction 

Cancer manifests as uncontrolled cell proliferation, followed by enhanced migration, invasion, and metastasis to other parts of the body. Numerous investigations support the role of cancer stem cells (CSCs) and their associated markers in tumour malignancies, for example, the cluster of differentiation 44 or CD44 [1]. As a non-kinase cell surface transmembrane glycoprotein that is overexpressed in CSCs and frequently undergoes alternative splicing to support cancer progression, it could severely influence treatment outcomes [2]. In the early 1980s, CD44 was first identified as a glycoprotein expressed on human [3] and murine mesenchymal cells [4]. Subsequently, it was cloned and classified as a cartilage link protein family member [5]. It is a single polypeptide chain encoded by a conserved gene located on either human chromosome 11 [6] or murine chromosome 2 [7] and is also known as In (Lu)-related protein p80, Pgp-1/Ly-24, ECMRIII, HUTCH-1, Hermes antigen, and importantly, hyaluronate receptor. Currently, CD44 is recognised as the main cell surface receptor for hyaluronate, which is the major extracellular matrix (ECM) component [8]. It is a member of the cell adhesion molecules (CAMs) family that plays important roles in cellular communication and adhesion between cells and the ECM [9]. In addition to its role in cellular adhesion and communication, it is essentially involved in several biological and functional processes, such as lymphopoiesis and myelopoiesis, leukocyte activation, angiogenesis and the release of cytokines, as well as many pathological processes including metastasis, epithelial–mesenchymal transition (EMT), cellular growth, proliferation, migration and invasion. Although abundant progress has been made regarding the structure and functional roles of CD44 and its diverse isoforms, the expression level associated with poorer clinicopathological impacts remains unknown. Thus, a significant area of study is aims to further define the functional roles of the different CD44 isoforms in various types of cancers. Increasing evidence suggests that some CD44 isoforms are promising prognostic biomarkers and therapeutic targets for many cancers. This review summarises current insights on CD44 structure and isoforms as well as the CD44-mediated oncogenic signalling pathway in several cancers. We also highlight CD44 interaction with many components in the tumour microenvironment and its functional roles and involvements in tumour progression and aggressiveness, as well as its clinical relevance and the possibility of targeting CD44 for cancer therapy. 

## 2. CD44 Structure and Isoforms

The full-length CD44 gene comprises 20 exons, with the constant exons 1–5 and 16–20 encoding the N-terminal and C-terminal domains respectively, which are homologous domains shared by all CD44 family members [10]. The smallest and the most expressed CD44 isoform is the CD44 standard (CD44s), constructed of ten constant exons with no variant exons [11]. The other isoform, the CD44 variant (CD44v), differs from CD44s by the insertion or excision of alternatively spliced exons between the N-terminal and C-terminal domains [12]. Tolg et al. [13] confirmed that besides ten constant exons, the mouse and rat genome has at least ten variant exons, all of which can be combined alternatively into CD44 mRNA. They suggested that the variant exons be numbered by the exon code v1 to v10. Screaton et al. [14] described the structure of the human CD44 gene, reporting that it contains 19 exons crossing some 50 kilobases of DNA with ten constant exons and nine variant exons coded v2–v10 [15,16]. CD44v isoforms may contain a single variant exon such as CD44v3 or CD44v6, or a combination of variant exons such as CD44v3–v7 and CD44v8-v10. Individual cells can continually alter the splicing of CD44 pre-mRNA, resulting in many possible combinations of these variant exons, giving the potential for great diversity [12].

**Figure 1:**
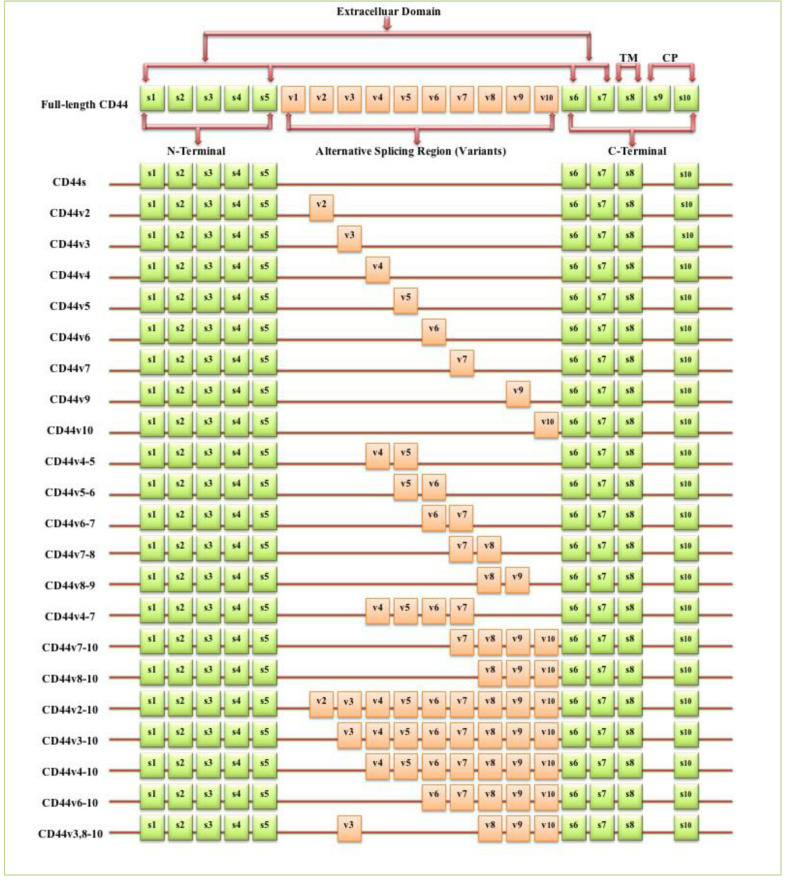
Schematic diagram of the mouse CD44 gene and most CD44 isoforms involved in cancer progression. The full-length CD44 gene contains 20 exons in mice and 19 exons in humans, with the constant exons 1–5 and 16–20 encoding the N-terminal and the C-terminal domains. CD44 standard (CD44s) is encoded by these ten constant exons and contains no variant exons, whereas the CD44 variant (CD44v) is produced by the alternative splicing of a variable insertion of nine extra exons in humans or ten extra exons in mice. These extra exons are exons 6-15, typically identified as (v1 to v10) in mice and the exons 7-15 identified as (v2 to v10) in humans and are located between the N-terminal and C-terminal domains. CD44v can contain one or multiple variant exons and exon 19 is spliced out in all CD44 isoforms. Abbreviations: CD44s, CD44 standard; CD44v, CD44 variant; s, standard; v, variant; TM, transmembrane; CP, cytoplasmic. Green boxes refer to the constant/standard exons. Orange boxes refer to the variant exons.

**Figure 2:**
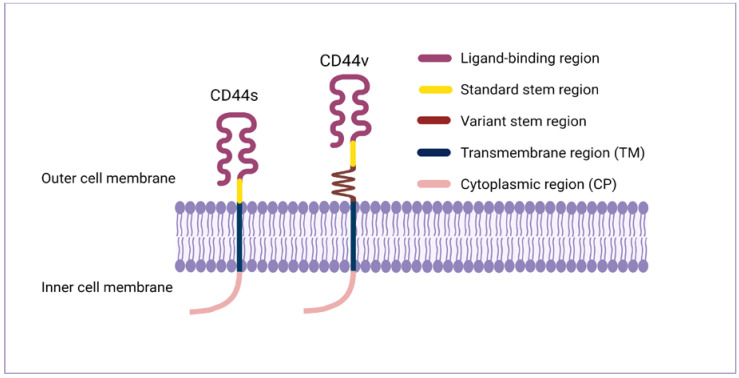
CD44 protein structure. The CD44 protein has four primary regions: the extracellular region consists of seven extracellular domains including N-terminal domains (ligand-binding region), the stem region (variable stem region and/or standard stem region) which is the alternative splicing area containing an insertion of one or more variant exons, the transmembrane region (TM), and the C-terminal cytoplasmic (CP) region.

The CD44 protein has four primary characteristic regions: the extracellular region, the stem region (standard stem region and/or variable stem region), the transmembrane region (TM), and the short C-terminal intracellular/cytoplasmic (CP) region [17]. The extracellular part consists of seven extracellular domains (1–5, 6 and 7 of the constant exons) including N-terminal domains (ligand-binding region). The stem region (alternative splicing area) has an insertion of one or more of the variant exons between exon 5 and exon 6. The transmembrane region is encoded by a single exon (exon 8), whereas the cytoplasmic region is encoded by exon 10 or exon 9. However, exon 9 is spliced out in almost all CD44 cDNA isoforms [12,18]. Several isoforms of the human CD44 molecule are associated with tumour progression and stemness in various cancers, such as breast cancer [19], gliomas [20,21], head and neck squamous cell carcinoma [22], pancreatic cancer [23,24], prostate cancer [25] and colorectal cancer [26,27] (Figure 1 and Table 1). The complexity of the CD44 protein is further augmented by post-translational modifications including variance glycosylation with O-glycans, N-glycans and glycosaminoglycans, such as chondroitin sulphate and heparan sulphate [17]. Due to these side-chain attachments, the conserved format of CD44 (37 kDa) is enlarged to 80–100 kDa with some isoforms surpassing 200 kDa due to a high level of glycosylation [12]. An illustration of CD44 protein structure is shown in Figure 2.

## 3. CD44 Expression in Normal Cells

CD44 is significantly expressed in lymphocytes, smooth muscle, fibroblasts and various types of epithelia and is involved in lymphocyte homing, cell adhesion and aggregation, cell migration, leukocyte activation, lymphopoiesis and myelopoiesis, angiogenesis and cytokine release [12,69]. CD44s was initially isolated from haematopoietic cells even though it is expressed in several other tissues including the liver, lung, pancreas, skin and central nervous system [12]. CD44s is expressed in adult tissues and embryo tissues from day 9.5 post coitum, whereas numerous isoforms of CD44v show a highly specialised expression pattern and are already in the egg cylinder at day 6.5 of development [70]. In contrast to CD44s, CD44v isoforms distribution is more restricted to a selected range of cells during specific stages of activation, maturation or development including macrophages, activated lymphocytes, keratinocytes and some epithelial cells such as in the stomach, bladder and uterine cervix [12] and many carcinomas. In normal tissues, CD44 isoforms play a role in hyaluronic acid (HA) metabolism regulation, whereby loss of CD44 expression disrupts HA metabolism and impairs hair regrowth, wound healing and keratinocyte proliferation [71].

## 4. CD44 Expression in Tumours

**Figure 3:**
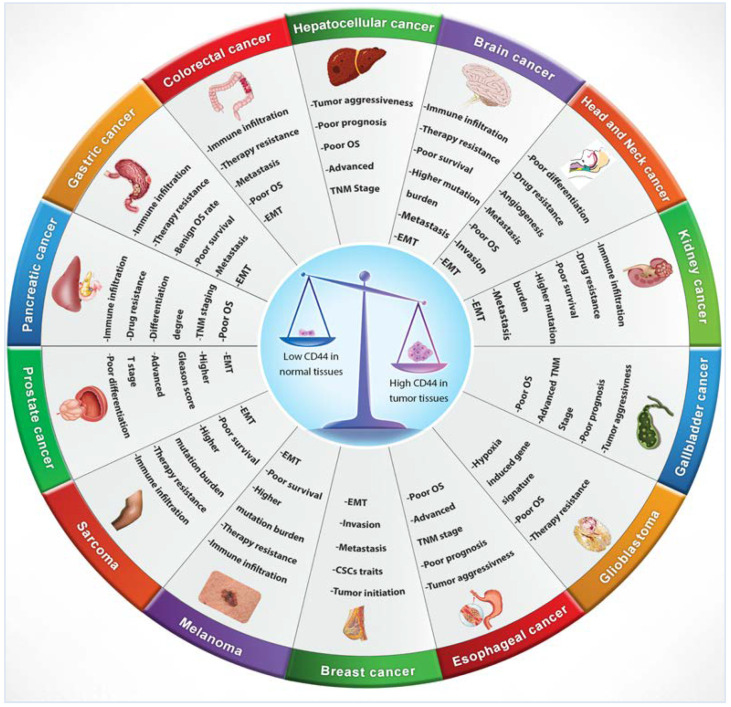
CD44 distribution in normal versus cancerous tissues and its correlation with clinical outcomes.

Numerous studies indicated that lymphoma, breast, colon and endometrial cancer have elevated levels of CD44 mRNA [69]. Increasing evidence also suggests that CD44 is extensively overexpressed in other cancer types including gallbladder, prostate, ovarian, oral squamous cell carcinoma and gastric cancer, correlating with aggressive biological behaviour and a poor prognosis [72]. The role of CD44 in tumours is not well defined, however, elevated levels of CD44 are associated with numerous malignant tumours. The physiological functions of CD44 indicate that it is involved in the metastasis of tumours [12]. For instance, lung adenocarcinoma cells show a high expression of CD44v, which correlates with enhanced CSCs characteristics, proliferation and resistance to chemotherapeutics [73], whereas these variants, especially CD44v6, are closely related to metastasis of pancreatic carcinoma cells [69]. Many studies have investigated CD44 expression levels in several cancers in comparison to their adjacent normal tissues and explored the relationship with tumour progression and clinicopathological outcomes by mining various publicly available databases, including The Cancer Genome Atlas (TCGA), Tumour Immune Estimation Resource (TIMER) database, Oncomine database, Gene Expression Profiling Interactive Analysis (GEPIA), In silico Transcriptomics (IST) database, R2 online database, SAGE Genie tools, and Human Gene Expression Map (HGEM) (Table 2 and Figure 3).

## 5. CD44s VS CD44v: Roles and Functions in Cancer Progression

The expression of CD44 isoforms within the tumour impacts key features of cancer cells such as tumourigenicity, tumour initiating potential, metastasis, chemo and/or radio-resistance, etc. Some researchers concluded that tumours expressing particular isoforms of CD44v are more aggressive compared to tumours expressing CD44s only. Recently, Zhang et al. [28] revealed that CD44s and not CD44v positively promotes tumour initiation and CSCs gene traits by activating the PDGFRβ/STAT3 cascade-signalling pathway. They showed that CD44s is the dominant isoform expressed in breast CSCs and that its elimination impaired CSCs signatures. Conversely, shifting alternative splicing from CD44v to CD44s by manipulating the splicing regulator ESRP1 led to an induction of CSCs traits. Brown et al. [30] also demonstrated that the isoform shift from the variant to the standard isoform was essential for breast cancer cells to undergo EMT, a process often activated during tumour metastasis and recurrence. CD44s accelerates EMT by activating AKT signalling, which results in the formation of EMT-associated recurrent tumours and apoptosis resistance in these tumours. Similarly, CD44s but not CD44v interacts with phosphorylated cortactin activated invadopodia, enabling breast tumour cells to degrade ECM and metastasise to distant organs such as the lungs. Depletion of CD44s eliminates invadopodia activity, prevents ECM degradation and reduces tumour cell invasion and metastasis [29]. CD44s is the predominant isoform highly expressed in hepatocellular carcinoma cells and its expression indicates poor survival in patients with hepatocellular carcinoma. CD44s but not CD44v regulates TGF-β signalling mediated mesenchymal phenotype, which is characterised by stimulated tumour invasiveness and increased expression of the EMT marker, vimentin and low E-cadherin expression [31].

In gallbladder cancer, Miwa et al. [32] found that CD44s^+^ cells highly expressed ZEB1 and ZEB2 transcription factors that mediate EMT and showed increased invasiveness, chemotaxis and distant metastasis compared to CD44v^+^ cells. However, CD44s^+^ cells exhibited decreased tumourigenicity while CD44v^+^ cells, particularly CD44v9, showed higher tumourigenicity suggesting that both CD44v and CD44s cells play different vital roles in tumour progression and metastasis. Other experimental studies have suggested that CD44v isoforms enhance tumour aggressiveness and bone metastasis in some cancers to a similar extent as CD44s. For instance, in breast and lung cancers, the introduction of ESRP1 prompted the splicing switch from CD44s to CD44v8-10 with no change in the total amount of CD44. This switching did not affect cell proliferation, invasion, migration, sphere formation or bone metastasis, demonstrating that the contribution of CD44v8-10 to tumorigenicity is comparable to that of CD44s [61]. While normal colonic mucosa does not express CD44 isoforms, its tumours present an extensive variation of CD44 isoforms. CD44v isoforms, especially CD44v3 and CD44v6, influence expressing metastasis genes and are involved in metastasis in colorectal cancer [40]. In the same type of cancer, CD44v2 was also highly expressed in both primary and xenografts tumours compared to the normal colonic mucosa, and this overexpression was associated with poor prognosis [36].

Meanwhile, CD44v8-10 was first defined in colon cancer cells, subsequently in gastric CSCs, and was discovered to be upregulated in primary and metastatic tumours [84]. CD44v8-10 expression, but not CD44s, marks gastric CSCs and enhances tumour initiation, probably through augmenting hypoxia or oxidative stress defence [62]. Likewise, CD44v5-6 and CD44v8-9, but not CD44s, are significantly increased in non-small cell lung adenocarcinoma and mediate tumour cell proliferation and poor prognosis by activating the KRAS/MAPK signalling pathway. KRAS-signalling induction additionally stimulates CD44 alternative splicing, resulting in a greater production of CD44v [58]. There are numerous reports that the expression of CD44v6 is an effective progression and prognosis marker in many cancers. For instance, CD44v6 is overexpressed in the mandibular invasive front of oral squamous carcinoma cells in patients with positive cervical lymph node metastasis and additionally, it is linked to the formation of tumour buds [43]. Yanamoto et al. [45] reported that a high intensity of CD44v6 in tongue squamous cell carcinoma was associated with local recurrence. In colorectal cancer, CD44v6 upregulation through nuclear β-catenin signalling activation contributed to tumour budding formation and the identification of those at high risk for locoregional failure in early staged tumours [46]. Ni et al. [44] demonstrated that in prostate cancer, CD44v6 is an important CSCs biomarker and is closely related to tumour cell proliferation, adhesion, invasion, metastasis, chemo- and radio-resistance, EMT induction, and the activation of PI3K/AKT/mTOR and Wnt signalling pathways. In contrast, another study of CD44 isoform expression in prostate cancer concluded that CD44s promoted tumour initiation, cell proliferation, invasion and migration, providing evidence of the correlation between total CD44 expression and prostate cancer progression for CD44s only. Furthermore, alterative splicing from CD44v to CD44s isoform enhanced tumour progression, EMT and stemness [33].

Amongst several molecules that have been extensively described and investigated for their possible roles in pancreatic carcinoma progression and tumourigenesis, CD44 is the most significant [47]. Li et al. [47] confirmed that decreased CD44s and increased CD44v expression in metastatic pancreatic carcinoma in three different cell lines and human tumour tissues. They also showed that CD44s^-^, CD44v6^+^ and CD44v9^+^ were significantly involved in the advanced TNM stage, liver metastasis and lymph node metastasis. In contrast, pancreatic ductal adenocarcinoma cells predominantly expressing high levels of CD44s are associated with an EMT phenotype, extremely invasive, develop gemcitabine resistance tumours and metastasise more rapidly [34]. Similarly, in pancreatic cancer, CD44s is involved in the radio-resistance of cancerous cells and strongly upregulated compared to CD44v after high-dose irradiation resulting in longer-term cell survival via the maintenance of ERK phosphorylation and radiation-stimulated EMT [35]. CD44v isoform expression is commonly believed to be more important for malignancy, especially when EMT is a vital step for metastasis, and is hence associated with a poorer prognosis [85]. However, and as discussed above, clinical data demonstrated that the CD44s isoform also enhanced tumour aggressiveness and metastasis in several cancers to a similar extent as CD44v. It is important to consider that all CD44 isoforms are prognosis markers and potential therapeutic targets for the prevention of metastasis.

## 6. CD44 Expression Regulation

**Figure 4:**
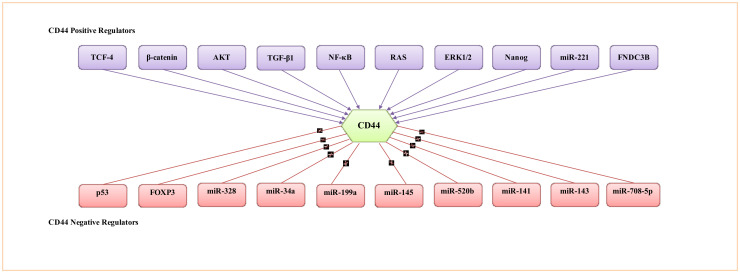
Representative transcription factors, protein kinases, cytokines and miRNA involved in the regulation of CD44 activity.

During tumour progression, CD44 isoform expression is regulated by numerous signalling networks. Since CD44 expression contributes to several signalling pathways involved in modulating multiple cellular processes that support tumourigenesis, it is vital to understand how CD44 expression is regulated and the key signalling networks involved in the regulation of CD44 expression. In this regard, several transcriptional factors, protein kinases, cytokines, epigenetic mechanisms and miRNAs are involved in the regulation of CD44 expression by acting as repressors or activators of CD44. A non-exhaustive list of mechanistic regulators is provided in Figure 4.

### 6.1. Regulation of CD44 by Transcriptional Factors, Protein Kinases and Cytokines

As CD44 overexpression is an early event in colorectal carcinoma, Wielenga et al. [86] established that CD44 is a target gene of Wnt/β-catenin in mice intestinal tumours, whereby the β-catenin/Tcf-4 signalling pathway mediates transcriptional upregulation of CD44 expression. Thereafter, CD44 expression upregulation by Wnt/β-catenin/Tcf-4 mediated transcription was also confirmed in human colorectal adenocarcinoma cells [87]. Smith et al. [88] demonstrated that the transcription factor (NF-κB) upregulates CD44 expression in triple-negative breast cancer cells, mainly via the interaction with the cis-regulatory element conserved region (CR1) located upstream of the CD44 promoter. The inhibition of NF-κB resulted in reduced CD44 expression. Wang et al. [89] found that CD44 expression was upregulated by activation of the β-catenin signalling pathway in mouse and human pancreatic ductal adenocarcinoma, resulting in EMT phenotype induction characterised by the upregulation of Zeb1 and Snail1 expression. Zhang et al. [28] described CD44s as predominantly expressing CD44 in breast CSCs and found that CD44s promoted CSCs signatures. Meanwhile, manipulating epithelial splicing regulatory protein 1 (ESRP1) suppresses CD44s-mediated induction of CSC traits. Godar et al. [90] showed that p53 inhibited CD44 expression by binding to a noncanonical p53 binding sequence in the CD44 promoter, and the p53 loss resulted in elevated CD44 levels which increased resistance to apoptosis in lung carcinoma cells. Forkhead box protein 3 (Foxp3) bound to the CD44 promoter and significantly inhibited its expression, suppressing the invasion and metastatic capabilities of human breast cancer cells [91]. In cervical cancer and breast cancer cells, the activation of β-catenin along with AKT signalling pathways were correlated with the upregulated expression of CD44. β-catenin knockdown and the inhibition of the AKT pathway significantly suppressed the expression of CD44 [92]. Cheng et al. [93] identified a positive feedback loop between CD44v6 isoforms and the RAS/MAPK signalling pathway. The RAS signalling pathway promoted CD44v6 production, which subsequently acted as co-receptors for several growth factors and tyrosine kinases to further activate RAS signalling. A study by Judd et al. [94] suggested that ERK1/2 signalling regulates CD44 expression in aggressive oral cancer cell lines and that the blockage of ERK1/2 decreased CD44 expression. Furthermore, Huang et al. [95] demonstrated for the first time that the ERK1/2-Nanog signalling pathway played a critical role in the maintenance of cells stemness and tumourigenic abilities by enhancing CD44^+^ CSCs in head and neck squamous cell carcinomas. Furthermore, Shang et al. [96] revealed that the transforming growth factor-beta 1 (TGF-β1) upregulated CD44 expression in prostate cancer cells. Similarly, crosstalk between the TGF-β pathway and CD44 expression upregulation was observed in oral and oesophageal cancer [97] and hepatocellular carcinoma [98].

### 6.2. Regulation of CD44 by Epigenetic Mechanisms

To determine whether CD44 expression might be associated with epigenetic regulation in lung cancer, an experiment using TGF-β treatment and DNA methyltransferase inhibitor (AZA) was performed. TGF-β treatment resulted in enhanced CD44 expression levels but AZA treatment did not stimulate CD44 expression, confirming that CD44 activated by TGF-β is not related to the epigenetic mechanism of aberrant promoter demethylation [99]. Other reports have suggested that CD44 expression levels can be epigenetically regulated by DNA methylation at the CD44 promoter region. Eberth et al. [100] showed that CD44 underwent de novo methylation in lymphoma cells, with the hypermethylation of CD44 resulting in transcriptional silencing of this gene, which can be reactivated by AZA treatment. Similarly, in neuroblastoma, Yan et al. [101] demonstrated that CD44 silencing was controlled by aberrant gene hypermethylation. Furthermore, in most prostate cancer cases, the loss of CD44 expression is associated with extensive hypermethylation of the CpG island CD44 promoter region [102,103]. Additionally, the CD44 gene in breast CSCs and CD44 gene hypomethylation was correlated with aggressive features of triple-negative breast cancer [104].

### 6.3. Regulation of CD44 by miRNAs

Several miRNAs were reported to regulate CD44 expression; for example, miR-328 is a potential regulator of CD44. Through studying the relative luciferase activity with diverse miR-328 mimic concentrations, a negative correlation between miR-328 and CD44 was established; CD44 constructs decreased as the concentration of miR-328 mimics increased [105]. Likewise, CD44 expression was diminished in gastrointestinal cancer cells forced to express miR-328, resulting in cancer cell growth inhibition and impaired resistance to chemotherapy and reactive oxygen species (ROS). In contrast, the stimulation of CD44 expression by a miR-328 inhibitor resulted in gastrointestinal cancer cell growth enhancement [106]. CD44 expression was also found to be suppressed by targeting the 3′-untranslated region (3′-UTR) of the CD44 gene, leading to perturbations of signalling pathways and the suppression of tumourigenesis and metastasis. For instance, miR-34a repressed CD44 expression in prostate CD44^+^ CSCs, resulting in metastasis and regeneration inhibition [107], and miR-199a-mediated targeting of CD44 in ovarian cancer-initiating cells suppressed tumourigenesis and multidrug resistance [108]. Similarly in gastric cancer, CD44 3′-UTR was found to be directly targeted by miR-145, with miR-145 overexpression repressing CD44 3′-UTR activity, which could be abrogated by blocking the miR-145/CD44 3′-UTR interaction, supporting significant augmentation of chemotherapy resistance and sphere formation [109]. CD44 was also identified as a direct molecular target of miR-520b, whereby miR-520b inhibited tumourigenesis of head and neck cancer through the regulation of cancer stemness conversion [110]. Another study demonstrated that miR-141 suppressed tumour growth and metastasis of prostate CSCs by targeting the CD44 gene [111]. In breast cancer cells, miR-143 inhibited the progression and stemness features by directly targeting CD44 3′-UTR [112]. In contrast, a positive relationship has been found between CD44 and miR-221, both of which are upregulated in hepatocellular carcinoma cells and tumours. Inhibition of miR-221 decreased CD44 protein expression, whereas the miR-221 mimic enhanced protein levels of CD44. Another study described a mechanism of miR-221-CD44 interaction involving the PI3K/AKT/mTOR pathway. Targeting the downstream effector of this pathway by the inhibition of miR-221 reduced CD44 expression [113]. CD44 is also regulated by a different class of non-coding RNA molecules known as the circular RNAs (circRNAs), single-stranded RNAs identified in malignant tumour cells which can join the 3′ end to the 5′ end of the RNA molecule. For example, the circFNDC3B regulates and increases the stability of CD44 expression by forming a ternary complex of circFNDC3B/IGF2BP3/CD44 mRNA, subsequently promoting cell invasion and migration of gastric cancer cells [114].

## 7. CD44-Downstream Signalling Pathways

**Figure 5:**
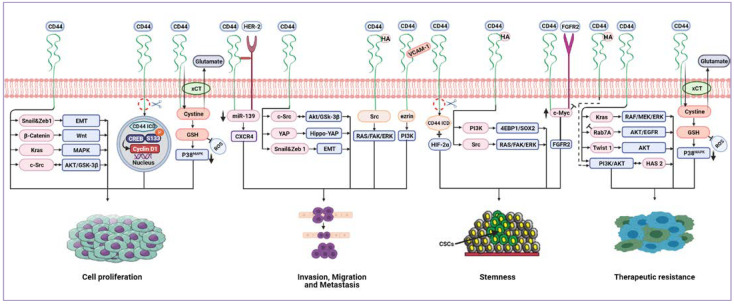
Cancer-associated signalling pathways modulated by CD44. CD44 can upregulate EMT biomarkers, Snail1 and Zeb1, promoting proliferation and invasion. CD44 activates KRAS through the MAPK pathway, hence promoting tumour cell proliferation and survival. CD44 mediates invasion and metastasis by binding to HER2, leading to the inhibition of miR-139 and upregulation of CXCR4. There is positive crosstalk between CD44 and FGFR2 to maintain cancer stemness. CD44 regulates tumour cell proliferation, invasion and migration by modulating c-Src via AKT/GSK-3β signalling. CD44/VCAM-1 interaction promotes invasion signalling by the ezrin/PI3K pathway. CD44 binds to HA, resulting in stemness development via the PI3K/4EBP1/SOX2 pathway. Also, the HA/CD44 interaction can drive tumour invasion, metastasis and stemness through Src activating RAS/FAK/ERK pathways. Similarly, HA/CD44 stimulates the PI3K/AKT signalling pathway to increase therapeutic resistance. CD44 can sustain EGFR and AKT signalling by inhibition of Rab7A, leading to therapeutic resistance. CD44 promotes the expression of HAS2 by activating the PI3K/AKT signalling pathway. HAS2 further enhances CD44-mediated PI3K/AKT signalling, thus creating a positive feedback loop that drives tumour cell resistance and survival. CD44 can stabilise the cystine/glutamate antiporter (xCT), leading to increased GSH and decreased ROS levels, which, in turn, results in tumour cell proliferation and therapeutic resistance mediated by suppression of the p38 pathway. CD44 regulates tumour cell proliferation by the Wnt/β-catenin signalling pathway. CD44 can promote invasion and migration through the activation of the Hippo-YAP oncogene signalling pathway. CD44 mediates tumour cells resistance by upregulating Twist1 and AKT signalling. CD44-ICD binds to CREB, enhances S133 phosphorylation and enriches CREB recruitment to the cyclin D1 promoter, thus promoting cyclin D1 activity, resulting in cell proliferation. CD44-ICD is released in a hypoxic environment and binds to HIF-2α leading to induced stemness. Abbreviations: EMT, epithelial–mesenchymal transition; HER2, human epidermal growth factor receptor 2; CXCR4, C-X-C chemokine receptor type 4; FGFR2, fibroblast growth factor receptor 2; EGFR, epidermal growth factor receptor; HAS2, hyaluronan synthase 2; GSH, glutathione; ROS, reactive oxygen species; CD44-ICD, CD44 intracellular domain; CREB, cAMP response element-binding; HIF-2α, hypoxia-inducible factors 2 alpha.

The activation of CD44 isoforms modulates the activities of the components of several signalling pathways including enzymes, protein kinase pathways, transcriptional factors and intracellular pathways to contribute to cancer cell proliferation, stemness, invasion, migration and metastasis, as well as drug resistance. Some of these signalling pathways are summarised in Figure 5.

In pancreatic intraepithelial neoplasia and pancreatic ductal adenocarcinoma, CD44 is central in promoting the upregulation of EMT biomarkers expression, Snail1 and Zeb1, as well as stimulating migration and invasion. CD44 knockdown using CD44 shRNA in pancreatic ductal adenocarcinoma cells not only efficiently reduced the expression of Zeb1 and Snail1 but also significantly impaired cell proliferation and invasion [89]. CD44 was found to critically contribute to activating the oncogenic KRAS signalling pathway through the MAPK pathway in lung adenocarcinoma cell lines, hence promoting tumour cell proliferation and survival [58]. CD44-mediated gastric cancer cell invasion and metastasis by binding to human epidermal growth factor receptor 2 (HER2) inhibits miR-139 and upregulates the miR-139 target gene, C-X-C chemokine receptor type 4 (CXCR4) [115]. Park et al. [116] demonstrated positive crosstalk between CD44 and fibroblast growth factor receptor 2 (FGFR2). While FGFR2 inhibits transcription of c-Myc, CD44 activates c-Myc expression, in turn enhancing FGFR2 transcription. The cooperative interaction of FGFR2 and CD44 maintains gastric cancer stemness via reciprocally regulating their expression by differentially regulating c-Myc transcription. CD44 in breast CSCs also played a pivotal role in the regulation of tumour cell proliferation, invasion and migration by modulating the levels of c-Src, a master regulator of the MAPK, PI3K, and STAT3 signalling pathways, via the inhibition of c-Jun and degradation by AKT/GSK-3β signalling [117]. In head and neck CSCs, by binding to HA, CD44 mediated stemness and CSCs fraction development via the PI3K/4EBP1/SOX2 pathway, while CD44/VCAM-1 interaction promoted invasion signalling by the ezrin/PI3K pathway [118]. CD44 can directly potentiate receptor tyrosine kinase (RTKs) signalling pathways and act as a coreceptor for several growth factors, such as tyrosine-protein kinase Met (c-Met), vascular endothelial growth factor receptor-2 (VEGFR-2) and epidermal growth factor receptor (EGFR), thus enhancing cancer cell proliferation and correlating with poor prognosis and metastatic potential [119]. Amongst CD44 isoforms, CD44v displays a greater affinity to HA compared to CD44s [120]. By activating RTKs signalling pathways, HA/CD44v6 interaction can drive tumour metastasis [121,122]. In colorectal cancer, this interaction facilitates colorectal CSCs colonisation, invasion and metastasis. Furthermore, the same interaction activates RAS and FAK pathways through Src, resulting in MAPK/ERK signalling pathway activation, and subsequently, enhanced cell proliferation. Similarly, the interaction between HA and CD44v6 stimulates the PI3K/AKT signalling pathway, thus increasing the resistance of colorectal cancer cells to apoptosis [123]. Additionally, in glioblastoma cells, CD44s attenuated EGFR degradation and sustained AKT signalling by the inhibition of Rab7A, which mediated EGFR trafficking for degradation, thus sustaining EGFR levels. CD44 depletion collectively with EGFR inhibition results in synergistic and robust glioblastoma cell death [124]. CD44s additionally promoted the expression of hyaluronan synthase 2 (HAS2) by activating the PI3K/AKT signalling pathway, which further enhanced CD44s-mediated PI3K/AKT signalling, thus creating a positive feedback loop to drive tumour cell survival in breast cancer cells [125]. Thanee et al. [126] revealed that CD44s and CD44v8-10 regulated redox status by stabilising cystine/glutamate antiporter (xCT), leading to increased glutathione (GSH) and subsequently, decreased ROS, which, in turn, suppressed the p38 MAPK pathway that correlates with poor prognosis for cholangiocarcinoma patients.

CD44 signalling plays a pivotal role in regulating the proliferation of chronic myeloid leukaemia cells by modulating the expression and activity of the Wnt/β-catenin signalling pathway. CD44 downregulation reduced β-catenin expression and augmented β-catenin phosphorylation, inhibiting cell proliferation [127]. Through the activation of the Hippo-YAP oncogene signalling pathway, CD44 promoted invasion and migration of docetaxel-resistant prostate cancer cells, which had a larger CD44^+^ population and positively correlated with poor survival of prostate cancer patients [128]. Zhang et al. [28] revealed that CD44s was preferentially and predominantly expressed in breast CSCs and promotes CSCs activities and tumour initiation through signal transducer and activator of transcription 3 (STAT3) via PDGFRβ/STAT3 signalling pathways. In hepatocellular carcinoma, CD44s also mediated tumour cells anoikis resistance and sphere formation capability by upregulating Twist1 and AKT signalling [129].

CD44 may undergo sequential proteolytic cleavage, releasing the intracellular domain CD44-ICD to translocate into the nucleus and transactivate gene expression. CD44-ICD bound to the cAMP response element-binding protein transcription factor (CREB-TF) and enhanced S133 phosphorylation and CREB-mediated gene transcription. CD44-ICD enriched CREB recruitment to the cyclin D1 promoter, thus promoting cyclin D1 activity, a regulator of protein transcription and cell proliferation, in thyroid carcinoma cells [130]. In glioma cells, CD44-ICD was released in a hypoxic environment and bound to hypoxia-inducible factors 2 alpha (HIF-2α) to induce stemness in glioma [131].

## 8. CD44 Receptor-Ligand Interactions

CD44 interacts with various ECM components, proteins and cytokines present in the tumour microenvironment. Interaction between ligands such as hyaluronan (HA), osteopontin (OPN) and matrix metalloproteinases (MMPs) with the CD44 receptor can stimulate several cellular signalling pathways [132]. CD44 functions in supporting tumour progression and aggressiveness can be attributed to its diverse binding ligands and interactome (Table 3).

HA is a protuberant molecule present in both the tumour stroma and the pericellular area surrounding tumour cells. It is a linear, negatively charged polysaccharide composed of repeating disaccharides of N-acetylglucosamine and glucuronate [133]. HA is produced by at least three essential intracellular plasma membrane proteins, HA synthase 1-3 (Has1-3) [134]. As the primary ligand of CD44, HA is a major CSCs-associated macromolecule involved in the regulation of cell stemness and drug resistance via the stimulation of EMT, ROS, secretion of extracellular vesicles/exosomes and epigenetic factors regulation (137]. In head and neck CSCs, HA binding to CD44 prompts upregulation of OCT4, Nanog and SOX2 expression, the hallmark of stem cell properties such as spheroid and clone formation, as well as cell growth, self-renewal, poor differentiation and additional chemoresistance in these head and neck CSCs [134].

In addition, HA interacts with CD44 to activate several oncogenic signalling pathways-associated cell surface receptors or domains, such as EGFR, c-Met, HER2, transforming growth factor-beta receptor type 1 (TGFβR1) and non-receptor kinases (Src family). This will consequently promote tumour growth via the activation of MAPK and PI3K/AKT signalling pathways as well as promoting chemoresistance, cell motility and metastasis-related pathways. For instance, HA binding to CD44 recruits RTKs, which in turn promotes cell survival and migration, conferring poor prognostic in pancreatic ductal adenocarcinomas [135]. In malignant pleural mesothelioma, HA and CD44 have been shown to ease cell motility, invasion and consequently, tumour progression [136].

In melanoma cells, the HA–CD44 axis increased cell proliferation [137] and promoted the expression of the inhibitor of differentiation/DNA binding (Id) proteins 1 and 3 via the BMP type II receptor (BMPR) to decrease OS in melanoma patients [138]. Depending on the CD44–HA interaction, liver cancer cells were found to roll on HA enriched endothelial cells, an essential step during metastasis [139]. In acute myeloid leukaemia cells, the binding of CD44 and HA enhanced cell proliferation, and blocking this binding significantly inhibited the growth of CD44^+^ leukaemia cells [140]. The HA–CD44 axis also activated ERM, ankyrin, Grb2, Gab-1 and Vav2, which drive cell migration via RAS, RhoA and Rac GTPase families [120,133,136,141]. The presence of HA in the tissue can enhance matrix stiffness, and in turn, conjointly modulate cell behaviour. High molecular weight HA concentration significantly impacted some biophysical matrix variations such as viscoelastic properties, specifically reducing the shear storage modulus and increasing compressive resistance [142]. As the inner tumour is disposed to compression, its stiffness is mainly determined by HA due to the fixed negative charges that create hydrated and gel-like regions within the tumour capable of resisting compressive stress [143]. An increase in HA accumulation can be found in advanced tumours and is frequently correlated with aggressive malignancy. Wound healing-associated myofibroblasts and activated cancer-associated fibroblasts (CAF) are presumed to be the main sources of HA in the range of 480 kDa for both cells. Tumours have been proposed to produce very low molecular weight HA, which is specifically regulated by CAF due to the high expression of HAS2 and hyaluronidase (HYAL1) in CAF, which might contribute to greater production of HA in the CAF matrix [144]. In contrast, high molecular mass HA accumulates in naked mole-rat tissues and protects against cancer. Once it is removed by either knocking down HAS2 or overexpressing the HA-degrading enzyme, HYAL2, naked mole-rat cells become susceptible to malignant transformation and easily form tumours [145,146]. The capacity of HA to interact with CD44 also depends on its molecular weight [147]. The amount of HA binding to CD44 increases as a function of HA size, with a half-maximal saturation at ~30 kDa. Furthermore, reversible interaction is confined to the smaller HA (< 10 kDa), whereas binding is irreversible with larger molecular weight HA (≥ 262 kDa) [148]. Recent work demonstrated that the direct correlation of the CD44–HA interaction on proliferation and invasiveness of melanoma cell lines is dependent on the molecular weight and the presentation form (matrix-bound or soluble) of HA. Only soluble low molecular weight HA (30-50 kDa) promoted cell proliferation and invasion in a CD44-dependent manner, while high molecular weight HA (500-750 kDa) and immobilised low molecular weight HA did not affect cell proliferation or invasion [149]. However, another study revealed that immobilised but not soluble HA enhanced co-localisation of CD44 and the receptor for hyaluronic acid-mediated motility (RHAMM), contributing to the aggressive and invasive phenotype in breast cancer cell lines [150].

Another essential ligand of CD44 is osteopontin (OPN), which is also known as early t lymphocyte activation 1 (eta-1), secreted phosphoprotein 1 (SPP1), and bone sialoprotein I (BSPI) [151]. OPN is the ECM component synthesised by osteoblasts, preosteoblasts and osteocytes, and is incorporated into the bone. Other than bone cells, OPN is also produced and secreted by odontoblasts and hypertrophic macrophages, brain, kidney, inner ear, and placenta cells [132,152,153]. The macrophage-secreted OPN in the tumour microenvironment binds to CD44 expressed by the tumour cells, which subsequently promotes clonal growth, invasion and metastasis. These effects require CD44 binding to TIAM1, which activates Rac1. Perturbing the OPN–CD44–TIAM1–Rac1 axis has been proposed as a therapeutic strategy to treat patients with metastatic bladder cancer [153]. Moreover, OPN and CD44 are highly expressed in hepatocellular carcinoma CSCs and are associated with increased incidence of tumour relapse and unfavourable prognosis [154]. Furthermore, the OPN–CD44 axis in hepatocellular carcinoma CD44^+^ CSCs mediated hepatitis C virus (HCV) replication and interferon (IFN) signalling as well as the expression of CSCs features, suggesting that this signalling pathway is critical for the propagation and pathogenesis of HCV in CSCs, contributing to CSCs phenotype maintenance and promoting aggressive tumour growth [155]. In addition to HA, as mentioned earlier, the dense stroma in pancreatic tumours is also rich in OPN, which sustains oncogenic signalling by interacting with CD44s and CD44v6 to enhance pancreatic cancer cells invasion [136,156]. Amongst the CD44 spliced variants, CD44v6 is highly expressed along with OPN in several cancers. Their interaction drives cancer progression and recurrence in ovarian cancer [157] and tumour cell migration in breast cancer [158]. Additionally, in colorectal cancer cell lines, OPN is overexpressed in response to hypoxic conditions and upregulates the expression of CD44s and its splice variants (specifically CD44v6), resulting in increased colorectal cancer cells radio-resistance [159]. Klement et al. [160] also revealed that both OPN and CD44 were highly expressed in human colon carcinoma compared to the normal colon. They discovered that the OPN/CD44 immune checkpoint controlled cytotoxic T lymphocytes (CD8^+^ T) cell activation and tumour immune evasion. Various isoforms of OPN have been identified to date, including OPN-a, OPN-b, and OPN-c, with OPN-a predominantly expressed in lung cancer. Through the CD44/NFκB pathway, OPN-a binds to, stabilises and activates CD44 expression, which in turn enhances lung cancer cell growth [161]. OPN also shares a perivascular expression pattern with CD44 in glioma CSCs and activates its signalling, increasing glioma aggressiveness, growth, stemness and radiation resistance [80]. Qiu et al. [162] indicated that the interaction of OPN and CD44 significantly promoted the progression and metastasis of advanced gastric cancer.

Matrix metalloproteinases (MMPs), another important CD44 ligand, are ubiquitously upregulated in many cancers and play important roles in promoting tumour angiogenesis, progression, invasion and metastasis [163]. MMP-14, a member of the MMPs family, has been shown to cleave CD44 to promote migration of osteosarcoma, pancreatic and breast cancer cells [164]. CD44 also induced EMT in colon carcinoma cells by upregulating MPP-14, stimulating cell invasion and migration [165]. In line with this, ovarian cancer patients who co-expressed both CD44 and MMP-14 had a poorer prognosis [166].

The CD44 signalling pathway can upregulate MMP-14 expression in basal-like breast cancers, whereby this upregulation correlates with the induction of basal-like breast cancer cells invasiveness [167]. The upregulation of MMP-2 and MMP-9 is associated with a poor prognosis in glioma and colorectal cancer patients [168,169,170]. Chetty et al. [171] reported that glioblastoma cells overexpressed CD44 and MMP-9 and their interaction controlled cell adhesion, invasion and migration. In non-small cell lung cancer, where CD44 was overexpressed [172], CD44s and MMP-2 co-expression was significantly associated with the lymph node metastasis, higher tumour TNM staging and poor patient prognosis [173]. MMP-2, together with MMP-9, participated in breast cancer cell invasion through their connection with CD44 [174]. The expression of MMP-9 and CD44 is also high in renal cell carcinoma. Through regulation by ribosomal S6 kinase 4 (RSK4), a downstream factor of the RAS/MEK/ERK signalling pathway, CD44 and MMP-9 overexpression is highly associated with the invasion and metastasis grade of metastatic clear cell renal cell carcinoma [175]. Expression of CD44 contributes to the enhanced bone metastasis in the human prostate cancer cell line PC3, grade IV prostate cancer, by promoting tumorigenicity, cell invasion, migration and HA production [176]. The role of CD44 in the invasion and migration of PC3 cells was demonstrated to be through MMP-9 activation. In the same cell line, the highly expressed runt-related transcription factor 2 (RUNX2) forms a complex with the overexpressed CD44, thereby activating many metastasis-related genes, including MMP-9, and consequently contributes to tumour sphere formation and migration [177].

**Table 3:** Summary of CD44 interactomes and their effects on cancer progression.

	Effect	Cancer Type	Reference
Hyaluronan (HA)	Stemness (spheroid and clone formation, self-renewal), cell growth, poor differentiation, chemoresistance	Head and neck CSCs	[134]
Hyaluronan (HA)	Cell survival, migration, poor prognosis	Pancreatic cancer	[135]
Hyaluronan (HA)	Cell motility, invasion, tumour progression	Pleural mesothelioma	[136]
Hyaluronan (HA)	Cell proliferation, poor survival	Melanoma	[137,138]
Hyaluronan (HA)	Cancer cells rolling, metastasis	Liver cancer	[139]
Hyaluronan (HA)	Cell proliferation	Acute myeloid leukaemia	[140]
Osteopontin (OPN)	Metastasis	Bladder cancer	[153]
Osteopontin (OPN)	Tumour growth, tumour recurrence, cell survival, metastasis, CSCs phenotype maintenance	Hepatocellular carcinoma	[154]
Osteopontin (OPN)	Invasion	Pancreatic cancer	[136,156]
Osteopontin (OPN)	Tumour progression and recurrence	Ovarian cancer	[157]
Osteopontin (OPN)	Migration	Breast cancer	[158]
Osteopontin (OPN)	Radio-resistance	Colorectal carcinoma	[159]
Osteopontin (OPN)	Tumour immune evasion	Colon carcinoma	[160]
Osteopontin (OPN)	Cell proliferation	Lung cancer	[161]
Osteopontin (OPN)	Aggressive growth, stemness, radio-resistance	Glioma CSCs	[80]
Osteopontin (OPN)	Tumour progression, metastasis	Gastric cancer	[162]
Matrix metalloproteinase14 (MMP-14)	Migration	Pancreatic cancer, breast cancer, osteosarcoma	[164]
Matrix metalloproteinase14 (MMP-14)	Invasion, migration	Colon carcinoma	[165]
Matrix metalloproteinase 14 (MMP-14)	Poor prognosis	Ovarian cancer	[166]
Matrix metalloproteinase 14 (MMP-14)	Poor prognosis, invasion	Breast cancer	[167]
Matrix metalloproteinase 9 (MMP-9)	Cell adhesion, invasion, migration	Glioblastoma	[171]
Matrix metalloproteinase 9 (MMP-9)	Invasion, metastasis stage	Renal carcinoma	[175]
Matrix metalloproteinase 9 (MMP-9)	Invasion, migration, sphere formation	Prostate cancer	[177]
Matrix metalloproteinase 2 (MMP-2)	Lymph node metastasis, histopathological grade, TNM stage, poor prognosis	Non-small cell lung cancer	[173]
Matrix metalloproteinase 2,9 (MMP-2), (MMP-9)	Invasion	Breast cancer	[174]

## 9. The Prognostic and Clinical Value of CD44 Expression in Advanced Cancer

Robust evidence supports that CD44 isoforms are closely related to the clinicopathological features of numerous cancers. CD44 is believed to undergo functional and structural alterations through malignant transformation, which contributes to the detachment of cancer cells from their original site, which then go on to invade the surrounding tissues. Immunohistochemistry analyses of CD44 expression in mucoepidermoid carcinoma revealed that high CD44 expression was significantly correlated with advanced tumours and increased recurrence or metastasis [178]. Similarly, there was a positive correlation between CD44 and tumour grade in salivary gland tumours [179]. Moreover, an analysis of human breast cancer tissues demonstrated that greater CD44 expression is linked to a higher histological tumour grade [180,181,182,183]. However, in patients with invasive breast cancer, CD44 expression was not associated with clinicopathological factors including histological grade, tumour size, tumour stage or metastasis status, except for one positive correlation with HER2 negative status [184]. Recent data by Roosta et al. [185] also demonstrated no relationship between CD44 expression and any clinicopathologic parameters in breast cancer, except with higher tumour stages. Likewise, a meta-analysis to address the prognostic significance of CD44 expression in breast cancer showed no noteworthy association between CD44 and OS or DFS [186]. Since the reported associations are diverse, additional studies with larger prospective cohorts are needed to further evaluate the association between CD44 expression and the prognosis of patients with breast cancer.

Moreover, high CD44 expression was also found in low-grade glioma and glioblastoma tissues compared to normal brain tissues, with the CD44 expression level serving as a predictive marker for OS rate in low-grade glioma patients [187]. Recently, Lee et al. [188] found that the expression of CD44 was positively correlated with a higher histological nuclear and shorter OS in clear renal cell carcinoma and nonclear renal cell carcinoma. Zanjani et al. [189] also reported that CD44 overexpression is statistically associated with more aggressive tumour behaviour, tumour grade and poor survival in clear renal cell carcinoma. However, in patients with chromophobe and papillary renal carcinoma cells, CD44 expression was not significantly correlated with prognosis. He et al. [72] reported that CD44 expression was significantly higher in gallbladder cancer patients with an advanced TNM stage, metastasised and poorly differentiated tumours. Moreover, a Kaplan–Meier analysis confirmed that the OS of patients with high CD44 expression was markedly poorer than those with low CD44 expression. Similarly in cholangiocarcinoma, the positive expression of CD44 was significantly related to large tumour size, high histologic grade, lymph node metastasis and distant metastasis. Taken together, these results demonstrate that the CD44-positive tumours suggest a poorer prognosis [190]. While some studies found no correlation between total CD44 and/or CD44v6 expression and clinical outcomes of patients with gastric cancer [191,192], conversely, the meta-analysis of Fang et al. [193] revealed that overexpression of total CD44 and/or CD44v6 is positively correlated with the TNM stage, T category, N category, invasion and distant metastasis. Moreover, their overexpression predicts a poor OS rate.

Many studies have also been conducted to investigate the relationship between CD44 expression and ovarian cancer progression and prognosis with contradictory results. Conic et al. [194] reported that low expression of CD44 was observed more frequently in advanced FIGO stage tumours and higher-grade tumours. Additionally, the mean survival was significantly longer in patients with high CD44 expression compared to those with low or absent CD44 expression. In the same cancer, CD44 overexpression was positively associated with progressive histologic grade and FIGO stage. In addition, multivariate analysis showed that the upregulation of CD44 was an independent predictive and prognostic factor for both OS and DFS of patients with ovarian cancer [195]. A recent meta-analysis also revealed that CD44 expression was significantly associated with a high TNM stage and poor OS in ovarian cancer patients [196]. CD44 is also a strong prognosticator of disease-specific survival (DSS) and nodal invasion in high-grade invasive urothelial carcinoma of the bladder when simultaneously expressed with fibroblast activation protein (FAP) [197]. The expression of CD44 in patients with pancreatic neuroendocrine tumours was positively related to poor tumour differentiation, high histological grade and an advanced stage. Survival analysis showed that CD44 was an important prognostic factor for OS and/or DFS. Moreover, in patients with no or low expression of CD44, a 100% DFS rate was observed, demonstrating a low recurrence risk [198]. In colorectal cancer, there was a strong statistically significant relationship between overexpressed CD44 in the primary colorectal carcinoma cell membrane and tumour grading, the degree of lymphocytic infiltration, lymphovascular invasion, peritumoral budding, lymph node ratio and lymph node metastasis status. CD44 was also correlated with OS reduction, representing an independent prognostic factor [199]. In oral cavity squamous cell carcinoma, some studies indicated no prognostic value of CD44 expression. For instance, Chen et al. [200] found no significant correlation between CD44 and T category, N category, tumour grade or survival. Another study revealed reduced CD44 expression in the advanced grades of oral cavity squamous carcinoma [201], which is in line with the data of Krump and Ehrmann [202]. However, numerous studies on oral carcinoma reported contradictory results, finding that CD44 overexpression was significantly associated with poorer histopathologic differentiation, higher tumour budding, invasion, lymph node status and metastasis. CD44 was also identified as an independent prognostic factor for poor OS, DSS and DFS in patients with advanced oral cancer [203]. High CD44 expression in oral squamous carcinoma cells is associated with increased depth of invasion (DOI), which predicts occult lymph node metastasis [204]. Another recent study revealed that CD44 overexpression was also associated with advanced T classification, lymphovascular invasion, perineural invasion, poor OS, DSS and recurrence-free survival (RFS) in patients with oral cancer [205].

**Figure 6:**
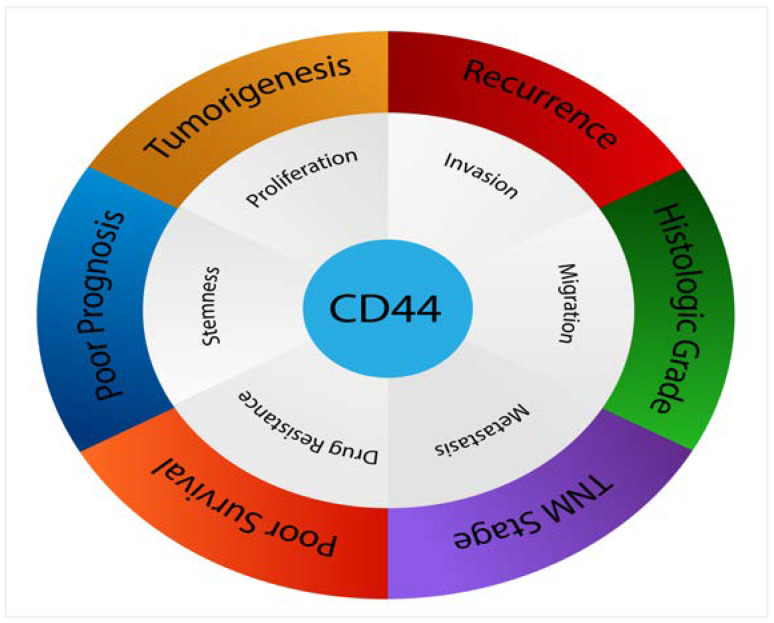
CD44 affects numerous pathological processes in cancers.

The conflicting data indicate that the role of CD44 is still controversial, with many authors arguing whether CD44 is significantly associated with poorer prognosis in many cancers including breast, gastric, ovarian and oral cancers. However, it seems that larger analyses demonstrate that increased CD44 expression has an unlimited correlation with higher histological tumour grade and an advanced clinical tumour stage. Overexpression of CD44 within numerous tumours, such as mucoepidermoid carcinoma, salivary gland tumours, breast cancer, gliomas, renal carcinoma, gallbladder cancer, cholangiocarcinoma, gastric cancer, ovarian cancer, bladder carcinoma, pancreatic tumours, colorectal cancer and oral cancer has been associated with increased tumorigenicity and decreased overall survival, indicating a poorer prognosis. Figure 6 presents the most relevant pathological features triggered by CD44 in various cancers.

## 10. Targeting CD44: A Promising Cancer Therapeutic Strategy

Targeted therapies are designed to specifically inhibit or block the aberrantly activated signalling pathways in tumour cells. Due to its widespread roles in promoting tumorigenesis, the inhibition of CD44 could impede tumour growth or sensitise tumour cells to therapy. CD44-targeted therapies include antibodies, peptides, pharmacological and natural inhibitors, HA-modified nanocarriers, siRNAs and CAR T cells therapy.

In the context of CD44-targeted antibody treatment, Arabi et al. [206] compared the antitumour activity of Doxil and monoclonal antibody (mAb)-modified Doxil against CD44, showing a significant improvement in cellular uptake of CD44-targeted, (mAb)-modified Doxil in CD44^+^ murine colon carcinoma cells compared to Doxil. Additionally, CD44-targeted, mAb-modified Doxil mice demonstrated a higher doxorubicin concentration inside the tumour cells compared to Doxil-treated mice. However, CD44^-^ mouse embryonic fibroblast cells showed similar uptake and cytotoxicity between the CD44-targeted, mAb-modified Doxil and Doxil treatments. Another antibody, the humanised mAb (RG7356), was shown to be cytotoxic in chronic lymphocytic leukaemia cells, especially the leukaemia B cells that overexpress CD44, and had little effect on normal B cells. Moreover, RG7356 induced rapid internalisation of CD44 in chronic lymphocytic leukaemia cells expressing the zeta-associated protein of 70 kDa (ZAP-70), resulting in ZAP-70 inhibition and subsequently promoting caspase-dependent apoptosis [207]. The transcriptomic profiling of human breast tumours and mouse stroma cells revealed that RG7356 induced a significant immune-stimulatory effect by binding to CD44^+^ tumour cells, resulting in the secretion of chemoattractants that are essential for immune cell recruitment (i.e., macrophages) to the tumour site, finally leading to antibody-dependent cellular phagocytosis (ADCP) of the cancerous cells by macrophages [208]. In the PC3 prostate cancer cell line, CD44 was discovered to carry the mAb F77 (a developed prostate cancer-specific mAb) epitope at the exon 14 region, in which F77 induced apoptosis in this cell line in a CD44-dependent manner. Meanwhile, CD44 knockdown almost completely inhibited F77-induced apoptosis [209]. In addition, the anti-CD44 mAb A3D8 enhanced apoptosis in acute myeloid leukaemia cells through caspase-8 activation by binding to CD44s protein [210]. In human ovarian cancer cell lines overexpressing CD44, the encapsulated glycosylated paclitaxel liposomes (gPTX-L) conjugated with anti-CD44 antibody efficiently enhanced cytotoxicity in vitro and in vivo, suppressing tumour growth in vivo [211].

In addition to antibody-mediated treatment strategy, potent synthetic peptides can also selectively bind to CD44 and serve as blocking agents. Peptides could be superior to antibodies for diagnostic and therapeutic purposes because of their robust physicochemical properties. Park et al. [212] developed a novel detection peptide as an alternative to antibodies for detecting CD44 in breast CSCs. They discovered seven different peptides (P1–P7) that bind comparably to CD44, in which P7 (FNLPLPSRPLLR) exhibited the highest specificity and affinity for CD44. Similarly, a polyvalent-directed peptide polymer (PDPP) was fabricated by conjugating the combinational peptides P6 and P7 on the poly-D-lysine (PDL) polymer to replace antibodies to recognise breast CSCs. PDPP had elevated affinity and thus inhibition potential against the CD44 biomarker in breast CSCs [213]. By targeting CD44, a short cationic antimicrobial peptide (CM11) loaded in HA/chitosan nanoparticles showed significantly higher cytotoxicity and apoptosis against several cancer cells including lung adenocarcinoma, neuroblastoma and pancreatic carcinoma cell lines compared to nanoparticles without HA coating [214].

Other than directly targeting CD44, several natural compounds and chemotherapeutic agents can indirectly inhibit the overexpressed CD44 isoforms in cancer cells and CSCs. Salinomycin (SLM) is a monocarboxylic polyether antibiotic isolated from *Streptomyces albus*, which, when incorporated with HA, targets and reduces the CD44^+^ CSCs population. Furthermore, a combination of HA-coated SLM nanoparticles and paclitaxel (PTX) nanoparticles showed higher cytotoxicity against CD44^+^ CSCs [215]. Also, sulfasalazine (SSZ), an inhibitor of the cystine-glutamate transporter subunit (xCT) interacts with CD44v and reduces the survival of human gastric CD44v^+^ CSCs both in vitro and in vivo [216]. Zerumbone (ZER), a monocyclic terpene derived from Southeast Asian ginger, suppressed CD44 expression in breast cancer cells through the inhibition of the STAT3 pathway [217]. Likewise, combined treatment of epigallocatechin gallate (EGCG) and curcumin-suppressed breast CSCs by reducing the CD44^+^ CSCs population and inhibiting STAT3 and NFκB signalling pathways [218]. Silibinin, a natural standardised extract of the milk thistle seeds, together with 5-fluorouracil (5-FU), inhibited CD44v6^+^ subpopulation proliferation in human colon carcinoma cells, and when CD44v6 was knocked down, cell sphere formation and migration were suppressed whereas apoptotic and autophagic cell death pathways were induced [219]. In glioblastoma cells, Galangin (3,5,7-trihydroxyflavone), a natural flavonoid in plants, was observed to inhibit CD44 and EMT through vascular endothelial growth factor (VEGF) downregulation, suppressing the proliferation, invasion migration and angiogenesis of tumour cells [220]. Apigenin (4′,5,7-trihydroxyflavone), another flavonoid compound present in many plants, induced apoptosis and inhibited prostate CD44^+^ CSCs and PC3 cell survival and migration mainly through the PI3K/AKT/NF-κB signalling pathway [221].

Researchers have taken advantage of the ability of HA to target and bind to CD44 in targeting CD44-overexpressing cancer cells. Eliaz and Szoka [222] provided evidence of the influential delivery of chemotherapeutic agents to cancer cells highly expressing CD44 by HA-modified liposomes. In this study, HA-targeted liposomes bound to the CD44^+^o-overexpressing B16F10 murine melanoma cell line but not to the CV-1 African green monkey kidney cell line, which expressed low levels of CD44. Moreover, doxorubicin (DOX), when encapsulated in HA-targeted liposomes, was significantly more potent than the nonencapsulated form in killing the cells, expressing high levels of CD44. Spadea et al. [223] evaluated the expression of CD44 isoforms and HA-internalisation efficacy in human dermal fibroblasts (HDFs) and cancer cell lines including prostate, thyroid, head and neck, breast, ovarian, pancreatic, colorectal and endometrial cancers. They found a positive correlation between the expression of CD44s and HA uptake level. Additionally, CD44s^+^ HDFs were less effective in the uptake of HA compared to CD44s^+^ cancer cells, indicating that HA targets CD44s expressed on cancer cells better than CD44s expressed on non-cancer cells. In gastric cancer, CD44 and HER2 are considered key molecules that participate in many crucial cellular processes. SN38 (7-ethyl-10-hydroxy-camptothecin) was successfully delivered to human gastric solid tumours through encapsulation in hybrid NPs comprised of a nanoparticle core made of PLGA and a lipoid shell synthesised by conjugating the AHNP peptides and *n*-hexadecylamine (HDA) to the carboxyl groups of HA. HA and AHNP on the nanoparticle surface allowed superior delivery of SN38 to gastric cancer cells by targeting CD44 and HER2, leading to repressed relative signalling cascades and inhibition of cell growth and invasion [224]. In addition, HA modification efficiently facilitated the delivery of curcumin (CUR)/DOX nanoparticles to hepatocellular carcinoma and human non-small cell lung cancer (NSCLC) for the treatment of multidrug resistance (MDR) cells through CD44 receptor-mediated targeted delivery [225].

Small interfering RNAs (siRNAs) are silencing RNAs that cause gene silencing through the repression of translation. A significant knockdown of CD44 expression was achieved by transfecting a designed siRNA into NSCLC cells. The inhibition of CD44 expression in these cells suppressed cell proliferation and colony formation ability [172]. Similarly, siRNA was used to inhibit CD44 expression in EGFR wild-type NSCLC cells and the downregulation of CD44 attenuated cell growth, promoted cell cycle arrest at the G0/G1 stage and stimulated cell apoptosis. Furthermore, CD44 inhibition significantly augmented the degradation of EGFR and enhanced the sensitivity of cells to cisplatin [226]. HA/PEI and HA/PEG nanoparticles were used to deliver multidrug resistance 1 (MDR1) siRNA in CD44^+^ ovarian cancer cells in combination with PTX, resulting in MDR1 downregulation, increasing apoptosis and the suppression of ovarian cancer growth [227]. In colon cancer, selective targeting of CD44^+^ cells was achieved via delivery of anti-KRAS siRNA loaded in poly hexamethylene biguanide (PHMB) and a chitosan complex coated with HA [228]. In the same cancer, CD44 was targeted directly by ON-TARGET plus human CD44 siRNA or indirectly by silencing mucin (MUC5AC) gene expression using a small hairpin RNA construct (pSUPER-Retro-shMUC5AC), resulting in decreased expression of CD44 cell migratory and invasion downstream signalling molecules, such as phosphorylated Src, AKT and integrin-β4 [229].

Recently, CD44 has been considered an attractive target for chimeric antigen receptor T cell (CAR T cells) therapy. Porcellini et al. [230] investigated the antitumour activity of the CD44v6-CAR T cells in some solid tumours broadly expressing CD44v6 in most cell lines including lung and ovarian carcinomas. They found that the generated CD44v6-CAR T cells controlled tumour growth and extend OS in the abovementioned two solid tumours in vivo. The antitumour activity of CD44-CAR T cells was also investigated for hepatocellular carcinoma in vitro and in vivo. CD44-CAR T cells had stronger tumour growth suppression capacity and prolonged survival in CD44^+^ hepatocellular carcinoma xenograft mice compared to normal and mock T cells [231]. The constructed bispecific tumour-targeted T cell engager (BiTE) molecule specific for CD44v6 was incorporated into an oncolytic helper binary adenovirus (CAdDuo) encoding an immune checkpoint blocker (PD-L1Ab) and immunostimulatory cytokine (interleukin [IL]-12) to form CAdTrio. This CD44-CAdTrio allowed HER2-CAR T cells to kill numerous CD44v6^+^ head and neck carcinoma cell lines and improve tumour control and survival [232]. A recent review by Alhabbab [233] concluded that the CAR T cells presently in use have a great success rate in leukaemia and, to some extent, in patients with solid tumours. However, according to the author, to date, no clinical trial has reported CD44-CAR T cells for the treatment of solid tumours. Based on these reports, CD44-CAR T cells induce remarkable tumour growth inhibition in several CD44-positive carcinoma cells and xenograft mice with no reported signs of CD44-CAR T cells mediating toxicity towards healthy tissues. Such findings sound promising; however, CD44-CAR T cell therapy has yet to be applied clinically. Since xenograft mice models do not fully reflect the nature of the human immune system, one major reason could be that CD44 is indeed expressed by many healthy cells, including T cells, fibroblasts and macrophages, and its targeting using CAR T cells might mediate toxicity in cancer patients.

## 11. Conclusions

Expansive evidence indicates that both CD44s and CD44v isoforms are overexpressed in a variety of cancers and regulated by several signalling networks. These isoforms play crucial roles in the development of various cancers, for instance, through their interaction with ligands such as hyaluronan, osteopontin and matrix metalloproteinases. Extensive studies revealed that both CD44s and CD44v play different vital roles in enhancing several carcinogenic processes including EMT, cell growth, proliferation, invasion, migration, metastasis, tumour initiation, stemness and therapeutic resistance. However, the conflicting data reported show that further investigation is required to explore what isoform has more impact on the key features of tumorigenicity and to define the fundamental mechanisms by which these isoforms promote tumorigenicity and tumour aggressiveness.

**Figure 7:**
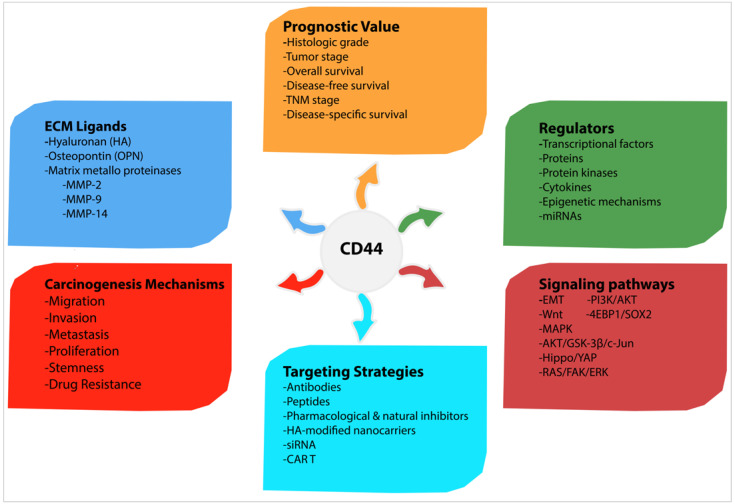
Summary of carcinogenic mechanisms and signalling pathways induced by CD44, as well as CD44 regulators, ligands, prognostic value and possible targeting strategies.

Regarding the conflicting reports, it is also debatable whether CD44 is significantly associated with a poorer prognosis in many cancers; however, more robust analyses demonstrate that increased CD44 expression has an unlimited correlation with higher histological tumour grade, advanced clinical tumour stage and shorter survival and indicates a poor prognosis. For these reasons, CD44 is a promising target for cancer therapy, particularly for tumours overexpressing CD44. Targeting CD44 isoforms may reverse some malignant behaviours and sensitise tumour cells to therapy. Current CD44-targeted therapies include antibodies, peptides, pharmacological and natural inhibitors, HA-modified nanocarriers, siRNAs and CAR T cells therapy. Despite the success of these approaches, along with mediated tumour growth inhibition in several CD44^+^ carcinoma cells and xenograft mice, they have yet to be translated into clinical application in human trials. Since xenograft mice models do not fully reflect the nature of the human body, one major reason could be that CD44 is indeed expressed by many healthy cells and its targeting might mediate toxicity in cancer patients. Accordingly, further analyses are still required before the translation to clinic trial. A summary of carcinogenic mechanisms and signalling pathways induced by CD44, as well as CD44 regulators, ligands, prognostic value and possible targeting strategies is shown in Figure 7.

## Figures and Tables

**Table 1:** CD44 isoforms relevant to cancer progression. Abbreviations: CSCs, cancer stem cells; EMT, epithelial–mesenchymal transition; DFS, disease-free survival; OS, overall survival; TNM stage, tumour (T), node (N), and metastasis (M) stage; FIGO stage, the international federation of gynaecology and obstetrics stage; NHL, Non-Hodgkin’s lymphoma; HPV, human papillomavirus; MAPK, mitogen-activated protein kinase.

CD44 Isoform	Association in Cancer Progress	Cancer Type	Ref
CD44, non-specified	Tumour cell aggregation, metastasis	Breast cancer	[19]
CD44, non-specified	Adhesion, migration, invasion	Glioblastoma	[20,21]
CD44, non-specified	Angiogenesis	Head and neck squamous carcinoma	[22]
CD44, non-specified	Invasion, metastasis, EMT, cancer progression, poor prognosis	Pancreatic cancer	[23,24]
CD44, non-specified	Proliferation, migration, invasion	Prostate Cancer	[25]
CD44, non-specified	Metastasis, poor differentiation, invasion	Colorectal cancer	[26,27]
CD44s	Tumour initiation, CSCs traits induction	Breast cancer	[28]
CD44s	Metastasis	Breast cancer	[29]
CD44s	EMT regulation, cancer progression	Breast cancer	[30]
CD44s	Poor DFS, poor OS, invasion, EMT	Hepatocellular carcinoma	[31]
CD44s	Invasion, metastasis, EMT, poor differentiation, chemotaxis	Gallbladder cancer	[32]
CD44s	Proliferation, invasion, migration, EMT, stemness	Prostate cancer	[33]
CD44s	EMT, invasion, metastasis, chemoresistance	Pancreatic ductal adenocarcinoma	[34]
CD44s	EMT, radio-resistance	Pancreatic cancer	[35]
CD44v2	Poor OS, advanced cancer stage	Colorectal cancer	[36]
CD44v2	Poor OS, invasion	Pancreatic cancer	[37]
CD44v3	Poor OS, invasion, metastasis	Oral squamous carcinoma	[38]
CD44v3	Stem cells self-renewal	Myeloid leukaemia	[39]
CD44v3	Metastasis	Colorectal adenocarcinoma	[40]
CD44v4	Proliferation, migration, radio-resistance	Head and neck squamous carcinoma	[41]
CD44v5	High histological grade, poor differentiation, poor OS	Hepatocellular carcinoma	[42]
CD44v6	Tumour budding, invasion, metastasis	Oral squamous carcinoma	[43]
CD44v6	Proliferation, invasion, adhesion, metastasis, EMT, chemo/radio-resistance	Prostate cancer	[44]
CD44v6	Local recurrence, invasion, metastasis	Tongue squamous carcinoma	[45]
CD44v6	Tumour budding, locoregional failure (metastasis, local recurrence)	Colorectal cancer	[46]
CD44v6	Proliferation, migration, radio-resistance	Head and neck squamous carcinoma	[41]
CD44v6	Metastasis	Colorectal adenocarcinoma	[40]
CD44v6	Poor OS, invasion	Pancreatic cancer	[37]
CD44v6	High histological grade, poor differentiation, poor OS	Hepatocellular carcinoma	[42]
CD44v6	Invasion, metastasis, poor OS, TNM stage	Pancreatic cancer	[47]
CD44v6	FIGO stage, poor prognosis	Cervical cancer	[48]
CD44v6	Metastasis, self-adhesion of aggressive NHL cells	Non-Hodgkin’s lymphoma	[49]
CD44v6	Infiltration, metastasis	Oesophageal squamous carcinoma	[50]
CD44v6	Proliferation, myofibroblastic differentiation	Gastric cancer	[51]
CD44v7	Proliferation, migration, radio-resistance	Head and neck squamous carcinoma	[41]
CD44v9	Increased tumourigenicity	Gallbladder cancer	[32]
CD44v9	Invasion, metastasis, poor OS, TNM stage	Pancreatic cancer	[47]
CD44v9	Proliferation, invasion, migration, EMT	Cholangiocarcinoma	[52]
CD44v9	Invasion, migration, worse prognosis	Bladder cancer	[53]
CD44v10	High histological grade, poor differentiation, poor OS	Hepatocellular carcinoma	[42]
CD44v10	Histological grade, clinical and pathological stage, poor survival	Renal carcinoma	[54]
CD44v10	Migration, metastasis, promote tumourigenesis	Breast cancer	[55,56]
CD44v4-5	Infiltration, metastasis	Oesophageal squamous carcinoma	[50]
CD44v4-5	Poor differentiation	Non-small cell lung carcinoma	[57]
CD44v5-6	Proliferation, KRAS/MAPK signalling, promoting tumour development	Lung adenocarcinoma	[58]
CD44v6-7	Metastasis	Pancreatic adenocarcinoma	[11]
CD44v7-8	High histological grade, poor differentiation, poor OS	Hepatocellular carcinoma	[42]
CD44v7-8	FIGO stage, poor prognosis	Cervical cancer	[48]
CD44v7-8	Invasion, high-risk HPV infection	Uterine cervical squamous carcinoma	[59]
CD44v8-9	Proliferation, KRAS/MAPK signalling, promoting tumour development	Lung adenocarcinoma	[58]
CD44v4-7	Metastasis	Pancreatic adenocarcinoma	[11]
CD44v7-10	Invasion	Prostate cancer	[60]
CD44v8-10	Migration, metastasis, sphere formation	Breast cancer	[61]
CD44v8-10	Tumour initiation, CSCs traits induction	Gastric cancer	[62]
CD44v8-10	Metastasis	Lung cancer	[63]
CD44v8-10	Metastasis, relapse	Gastric cancer	[64]
CD44v8-10	Poor prognosis, chemo/radio-resistance	Oesophageal squamous carcinoma	[65]
CD44v8-10	Chemoresistance	Urothelial cancer	[66]
CD44v2-10	CSCs traits induction, tumour subtype, oncogenic signalling pathways	Breast cancer	[67]
CD44v3-10	CSCs traits induction, tumour subtype, oncogenic signalling pathways	Breast cancer	[67]
CD44v3-10	Metastasis, self-adhesion of aggressive NHL cells	Non-Hodgkin’s lymphoma	[49]
CD44v4-10	Tumour initiation, wild-type phenotype	Intestinal cancer	[15]
CD44v6-10	Metastasis, self-adhesion of aggressive NHL cells	Non-Hodgkin’s lymphoma	[49]
CD44v6-10	Metastasis, relapse	Gastric cancer	[64]
CD44v3, 8-10	Metastasis, relapse	Gastric cancer	[64]
CD44v3, 8-10	Metastasis, migration	Breast cancer	[68]

**Table 2:** Low and high CD44 expression in normal and tumour tissues respectively and association with clinical outcomes.

Cancer Type	Correlation with Clinical Outcomes	Public Database	Reference
Gallbladder cancer, hepatocellular carcinoma, cholangiocarcinoma	Poor prognosis, advanced TNM stage, poor OS, aggressive tumour behaviour (proliferation, migration, invasion, clonogenicity)	TCGA database	[72]
Colon cancer, gastric cancer, brain cancer, stomach cancer, pancreatic cancer, liver cancer	Benign OS rate in gastric cancer, poor OS in colon cancer, TNM staging, differentiation degree, and poor survival in pancreatic cancer	SAGE Genie and Oncomine database	[74,75]
Head and neck squamous carcinoma	Poor OS, poor differentiation, angiogenesis, immune regulation, invasion	TCGA database	[76]
Head and neck squamous carcinoma	Pro-angiogenetic phenotype	TCGA database	[22]
Prostate cancer	Advanced T stage, higher Gleason score, poor differentiation	TCGA database	[77]
Colon adenocarcinoma	Therapy resistance	TCGA database and GEPIA	[78]
Head and neck squamous carcinoma, acute myeloid leukaemia (AML), lung carcinoma	Not specified	IST database and HGEM database	[79]
Glioblastoma	Poor OS, hypoxia-induced gene signature	TCGA database	[80]
Glioblastoma	Poor OS, therapy resistance	R2 online database	[81]
Invasive ductal breast carcinoma	Invasion, metastasis	TCGA database	[82]
Brain and CNS cancer, colorectal cancer, melanoma, sarcoma, gastric cancer, head and neck carcinoma, kidney cancer, oesophageal cancer, cholangiocarcinoma, pancreatic cancer	EMT, drug resistance, metastasis, immune infiltration and suppression features, poor survival, higher mutation burden, afflict older patients	Oncomine database and TIMER database	[83]
